# Association Between Local Anesthetic Volume–Dose Combinations and Optic Nerve Sheath Diameter as an Indirect Marker of Intracranial Pressure During Ultrasound-Guided Supraclavicular Brachial Plexus Block: A Randomized Trial

**DOI:** 10.3390/medicina62061103

**Published:** 2026-06-05

**Authors:** İsmet Çopur, Rıza Hakan Erbay, Seher İlhan, Turan Evran

**Affiliations:** Department of Anesthesiology and Reanimation, Faculty of Medicine, Pamukkale University, Denizli 20700, Turkey; icopur@pau.edu.tr (İ.Ç.); rherbay@pau.edu.tr (R.H.E.); seheri@pau.edu.tr (S.İ.)

**Keywords:** supraclavicular brachial plexus block, optic nerve sheath diameter, intracranial pressure, local anesthetic volume–dose, ultrasound-guided nerve block, regional anesthesia

## Abstract

*Background and Objectives*: This prospective, randomized study aimed to evaluate the effects of different local anesthetic (LA) volume–dose combinations administered during supraclavicular brachial plexus block (SCBPB), a widely used technique in upper extremity surgery. These effects were assessed by analyzing changes in the ratios of optic nerve sheath diameter (ONSD) to eyeball transverse diameter (ETD), obtained by ultrasound (US) and considered indirect measures of intracranial pressure (ICP). *Materials and Methods*: Sixty four ASA I–II patients aged 18–50 years undergoing upper extremity surgery were randomized into four groups receiving 15 mL (Group A), 20 mL (Group B), 25 mL (Group C), or 30 mL (Group D) of LA (equal volumes of 0.5% bupivacaine and 2% prilocaine). ONSD/ETD ratios were measured bilaterally at baseline, 20, and 60 min. Perfusion index (PI), end-tidal carbon dioxide (EtCO_2_), block onset times and block duration were also assessed. *Results*: Groups C and D showed significant bilateral increases in both ONSDint/ETD and ONSDext/ETD ratios at 20 and 60 min compared with baseline (*p* < 0.05). Group B demonstrated a significant increase only in the ONSDext/ETD ratio on the block side, whereas Group A showed no significant change. PI increased earlier and more markedly with increasing LA volume–dose. No significant intergroup differences were observed in EtCO_2_. In pairwise comparisons, sensory block onset was significantly longer in Group A than in Groups B, C, and D (*p* < 0.001). Motor block onset was significantly longer in Group A than in Groups C and D, and in Group B than in Group D (*p* < 0.001). Analgesia duration was significantly shorter in Group A than in Groups B, C, and D, and in Group B than in Groups C and D (*p* < 0.001). *Conclusions*: Increasing the LA volume–dose in US-guided SCBPB accelerates sensory and motor block onset and significantly prolongs block duration. A volume-dependent increase in ONSD/ETD ratios was observed on both the blocked and contralateral sides. PI showed an early and marked increase, particularly in high-volume–dose administrations, reflecting block success. Non-invasive EtCO_2_ monitoring did not detect significant changes.

## 1. Introduction

The supraclavicular brachial plexus block (SCBPB) is a peripheral nerve block technique used in upper extremity surgery that provides sensory and motor blockade by blocking the trunks of the brachial plexus at the supraclavicular level [1]. Traditionally performed using anatomical landmarks and nerve stimulation techniques, this block has in recent years been performed more safely and effectively under ultrasound (US) guidance [2,3]. The use of US allows direct visualization of the brachial plexus, the surrounding vascular structures, and the pleura, enabling real-time monitoring of local anesthetic (LA) spread, which increases block success and reduces the incidence of complications [4,5]. Although SCBPB is considered a safe technique, procedure-related complications such as pneumothorax, hemothorax, diaphragmatic paralysis, Horner’s syndrome, and local anesthetic toxicity have been reported [6,7]. In particular, it has been reported that higher LA volumes in SCBPB may be associated with an increased incidence of side effects and complications, owing to greater cephalad spread compared with lower volumes [8]. Similarly, it has been suggested that cephalad spread in other upper extremity blocks may also exert effects on cranial physiology and lead to an increase in intracranial pressure (ICP). Notably, an increase in the optic nerve sheath diameter (ONSD) has been demonstrated following cervical sympathetic block, suggesting that cervical sympathetic involvement may contribute to changes in ICP [9]. In addition, diaphragmatic dysfunction secondary to phrenic nerve involvement has been reported following SCBPB [10]. It has been suggested that the consequent increase in PaCO_2_/EtCO_2_ may contribute to a rise in ICP through cerebral vasodilation [11]. Another study on interscalene block reported that LA volume may contribute to elevated ICP by reducing venous return through extravascular compression of the internal jugular vein. This increase was indirectly assessed via US-measured ONSD [12]. ONSD measurement is a method used for the noninvasive assessment of ICP, owing to the sensitivity of the subarachnoid space surrounding the optic nerve to changes in ICP [13]. Systematic reviews and meta-analyses have reported that US-measured ONSD has high sensitivity and specificity for detecting elevated ICP [14]. Studies in the literature investigating the effects of brachial plexus blocks on ICP through ONSD measurements are limited. This study aimed to evaluate the effects of different LA volume–dose combinations on ICP during US-guided SCBPB through ONSD measurements. We hypothesized that an increase in LA volume–dose would lead to an increase in ONSD.

## 2. Materials and Methods

### 2.1. Study Design

This study was designed as a prospective, parallel-group randomized trial with a 1:1:1:1 allocation ratio and an exploratory framework at Pamukkale University Hospital and was registered at ClinicalTrials.gov on 14 May 2024 (NCT06428461; https://clinicaltrials.gov/study/NCT06428461). Ethical approval was obtained from the Clinical Research Ethics Committee of Pamukkale University (E-60116787-020-512860). The study was conducted in accordance with the Declaration of Helsinki. No important changes were made to the trial methods, outcomes, or analyses after trial commencement. Patients and/or the public were not involved in the design, or conduct, or reporting, or dissemination plans of this research.

### 2.2. Inclusion Criteria

The study enrolled patients aged 18–50 years with American Society of Anesthesiologists (ASA) physical status I–II scheduled for upper extremity surgery at Pamukkale University Faculty of Medicine Hospital.

### 2.3. Exclusion Criteria

Exclusion criteria were as follows: any condition known to elevate ICP; renal, hepatic, or cardiac failure; second-degree or third-degree atrioventricular block; unstable angina; a history of myocardial infarction within the previous 6 weeks; a heart rate <50 bpm; a systolic blood pressure <90 mmHg; chronic obstructive pulmonary disease; chronic asthma; neurological or psychiatric disorders; allergy to the study drugs; pregnancy; anatomical contraindications to SCBPB; and refusal of the anesthetic technique.

### 2.4. Randomization and Grouping

Sixty-four patients were randomly allocated into four equal groups (n = 16 per group) using a simple, computer-generated randomization sequence (Research Randomizer; www.randomizer.org). Allocation concealment was achieved using sequentially numbered, opaque, sealed envelopes that were opened upon arrival in the operating room. SCBPB was performed by a single anesthesiologist according to the assigned group. This study was designed as a triple-blind randomized controlled trial in which the patients, the outcome assessor, and the data analyst were blinded to group allocation. Due to the nature of the intervention, the anesthesiologist performing the block was necessarily aware of the administered LA volume–dose; however, the same anesthesiologist was not involved in measurements, data collection, outcome assessment, or data analysis. ONSD measurements were performed by a second anesthesiologist, independent of the anesthesiologist performing the block, who was blinded to both group allocation and the administered LA volume–dose. Patients were also blinded to their group allocation. During the statistical analysis process, the groups were coded as A–D, and all analyses were conducted by an independent investigator blinded to the intervention allocation. The groups were defined according to the administered LA volume–dose: Group A (15 mL, n = 16), Group B (20 mL, n = 16), Group C (25 mL, n = 16), and Group D (30 mL, n = 16). The LA mixture consisted of equal volumes of 0.5% bupivacaine (Buvicaine^®^, Polifarma, Kapaklı/Tekirdağ, Turkey) and 2% prilocaine (Priloc^®^, Vem, Kapaklı/Tekirdağ, Turkey). The LA concentration was kept constant across all four groups; consequently, the total administered dose increased proportionally with the injected volume.

### 2.5. Study Protocol

**Figure 1:**
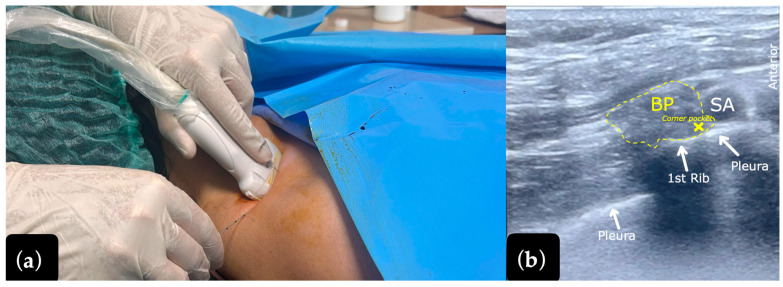
SCBPB. (**a**) In-plane positioning of the probe and needle in the supraclavicular fossa. (**b**) US image. BP: Brachial plexus SA: Subclavian artery.

Upon arrival in the operating theatre, standard ASA monitoring was established, including continuous peripheral oxygen saturation (SpO_2_), electrocardiography, heart rate, respiratory rate, end-tidal carbon dioxide (EtCO_2_; Capnostream 35, Medtronic, Ireland), noninvasive blood pressure, and body temperature. For premedication, all patients received 1 mg of intravenous midazolam together with supplemental oxygen at 1–2 L/min. Patients were placed in the supine position for SCBPB. All SCBPBs were performed by a single experienced anesthesiologist via a midclavicular entry point using a LOGIQ e portable ultrasound system (GE HealthCare, Chicago, IL, USA) equipped with a 12L-RS high-frequency linear array transducer (4.2–13.0 MHz), in conjunction with a nerve stimulator (Plexygon, Vygon, Padova, Italy) and a 21-gauge, 100 mm echogenic stimulating needle (Echoplex+, Vygon, Ecouen, France). All ONSD and ETD measurements were performed by a single anesthesiologist experienced in optic nerve sheath diameter ultrasonography, who was blinded to group allocation. Measurements were conducted using a standardized ultrasound protocol at 3 mm posterior to the globe in both transverse and sagittal planes. At each time point (0, 20, and 60 min), measurements were repeated at least twice, and the mean values of the transverse and sagittal measurements were used for analysis. SCBPB was performed by injecting LA into the corner pocket adjacent to the brachial plexus and subclavian artery (Figure 1). Sensory block was evaluated using the pinprick test, and motor block was assessed using the modified Bromage scale (0 = no block to 3 = complete block). Assessments were initiated 5 min after block placement and repeated at 5 min intervals. Sensory block onset was defined as the time point at which the pinprick test became negative. Motor block onset was defined as the time from completion of local anesthetic injection to the achievement of a modified Bromage score ≥2. Duration of analgesia was defined as the time from completion of the block to the first request for analgesia or the first report of pain requiring analgesic intervention. The perfusion index (PI) was measured and recorded from both the blocked and contralateral arms at 0, 2, 4, 6, 8, 10, 15, 20, and 25 min using a bedside monitor (uMEC 12, Mindray, Shenzhen, China). The time at which PI reached three times its baseline value (PI_3x_) was recorded in minutes, in line with previous studies regarding this parameter as an early indicator of block success [15]. When additional analgesia was required during surgery, LA was infiltrated into the surgical site by the surgeon, and the administered volume was recorded. In cases where sedoanalgesia was insufficient, conversion to general anesthesia was planned. Adverse events were assessed by routine clinical observation during the intraoperative and postoperative follow-up periods. Assessed events included pneumothorax, hemothorax, Horner’s syndrome, hemidiaphragmatic paralysis, local anesthetic systemic toxicity, vascular puncture, and neurological complications.

### 2.6. ONSD and ETD Measurement Technique

**Figure 2:**
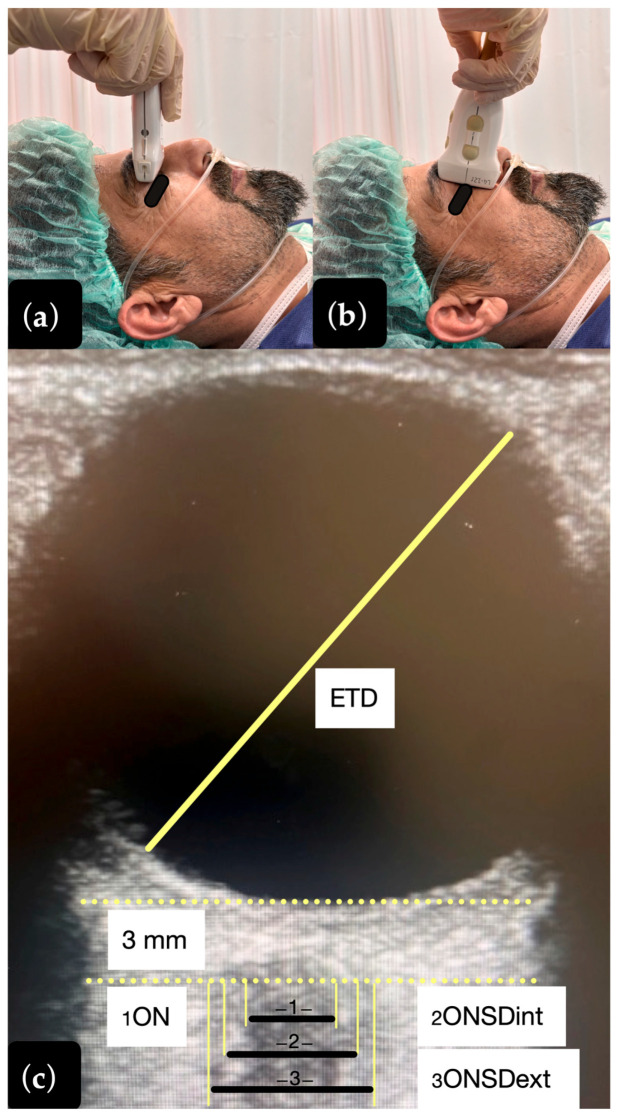
Ultrasound measurement. (**a**) Transverse and (**b**) sagittal probe positions. (**c**) B-mode image showing ETD, ONSDint and ONSDext. ONSD was measured at 3 mm posterior to the papilla.

Before block placement, with the patient in the supine position, the eyeball transverse diameter (ETD), internal optic nerve sheath diameter (ONSDint), and external optic nerve sheath diameter (ONSDext) were measured in both eyes using B-mode ultrasonography on the LOGIQ e system (GE HealthCare, Chicago, IL, USA) with a 12L-RS high-frequency linear array transducer (4.2–13.0 MHz). All ONSD and ETD measurements were performed by a single anesthesiologist experienced in optic nerve sheath diameter ultrasonography. Measurements were conducted using a standardized ultrasound protocol at 3 mm posterior to the globe in both transverse and sagittal planes. At each time point (0, 20, and 60 min), measurements were repeated at least twice, and the mean values of the transverse and sagittal measurements were used for analysis. ONSDint was defined as the distance between the inner borders of the optic nerve sheath (pia mater boundary), whereas ONSDext was defined as the distance between the outer borders (dura mater boundary). ETD was defined as the maximal retina-to-retina distance at the widest cross-sectional diameter of the globe, in accordance with previously described ultrasonographic methodology [16,17]. The measurement was performed based on the clearest visualization of the retinal boundaries rather than a fixed horizontal axis, which may result in a slightly oblique orientation (Figure 2).

### 2.7. Outcomes

The primary outcome of the study was the change in ONSD measured bilaterally at baseline and at 20 and 60 min after SCBPB. Secondary outcomes were the temporal changes in PI and noninvasive EtCO_2_, the sensory and motor block onset times (min), and the volume of additional LA required intraoperatively (mL). Postoperative pain was assessed using the Verbal Pain Score (VPS) at 0, 1, 2, 4, 6, 12, and 24 h after surgery.

### 2.8. Data Analysis and Statistical Methods

All statistical analyses were performed using IBM SPSS Statistics (v31.0 IBM Corp., Armonk, NY, USA). Continuous variables were presented as mean ± standard deviation (SD) for clinical interpretability, and categorical variables as numbers (n) and percentages (%). The normality of the data distribution was assessed using the Shapiro–Wilk test. Because the assumptions of parametric tests were not met, between-group comparisons were performed using the Kruskal–Wallis H test. When a significant difference was detected, pairwise comparisons were performed using the Mann–Whitney U test, with *p* values adjusted by the Bonferroni correction (α = 0.05/6 = 0.0083). Within-group repeated measures were analyzed using the Friedman test, followed by the Wilcoxon signed-rank test with Bonferroni correction (α = 0.05/3 = 0.0167) for post hoc pairwise comparisons. Categorical variables were compared using the chi-square (χ^2^) test. A two-sided *p* value < 0.05 was considered statistically significant.

Sample size calculation. The sample size was calculated based on the primary outcome, the change in ONSD. Based on a large effect size (f = 0.5; converted from Cohen’s d = 1.147) reported for ONSD changes in previous studies [9,12], a minimum of 64 patients (16 per group) was required to detect a between-group difference among the four groups with 90% power and a two-sided α of 0.05 using a one-way ANOVA approach. The calculation was performed using G*Power (v3.1.9.7, Heinrich-Heine-Universität, Düsseldorf, Germany). No interim analyses or stopping rules were applied. Median differences with 95% confidence intervals were calculated using the Hodges–Lehmann approach.

## 3. Results

**Figure 3:**
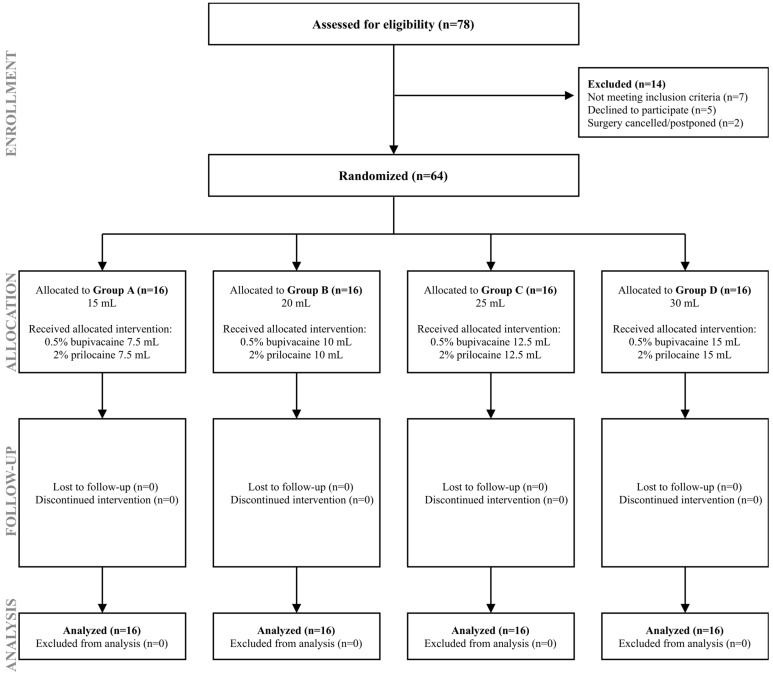
Flowchart of patient enrollment, randomization, and follow-up.

Between 20 May 2024 and 3 August 2024, a total of 78 patients were assessed for eligibility. Of these, 14 patients were excluded: 7 did not meet the inclusion criteria, 5 declined to participate, and the surgeries of 2 were cancelled or postponed. The remaining 64 eligible patients were randomized into four groups, with 16 patients per group, and included in the study (Figure 3).

Baseline demographic and clinical characteristics were well balanced across the four groups (Table 1).

At baseline, no significant between-group differences were observed in ONSDint/ETD or ONSDext/ETD ratios on either the block side or the contralateral side (*p* > 0.05). On the block side, significant intergroup differences in the ONSDint/ETD ratio were detected at 20 and 60 min (*p* = 0.002 and *p* = 0.001), and in the ONSDext/ETD ratio at both time points (*p* < 0.001) (Table 2). In intragroup time analyses, Groups C and D showed significant changes over time in both parameters (ONSDint/ETD: *p* = 0.008 and *p* = 0.001; ONSDext/ETD: *p* < 0.001), whereas Group B showed a significant change over time only in the ONSDext/ETD ratio (*p* = 0.015). Absolute ONSD values and their changes from baseline for all groups and time points are provided in the Supplementary Material Table S1.

Similarly, significant intergroup differences on the contralateral side were observed in both ratios at 20 and 60 min (ONSDint/ETD: *p* = 0.001; ONSDext/ETD: *p* < 0.001). In intragroup time analyses, Groups C and D showed significant changes over time in both parameters (ONSDint/ETD: *p* = 0.002 and *p* = 0.001; ONSDext/ETD: *p* = 0.013 and *p* < 0.001), whereas Groups A and B showed no significant changes.

**Figure 4:**
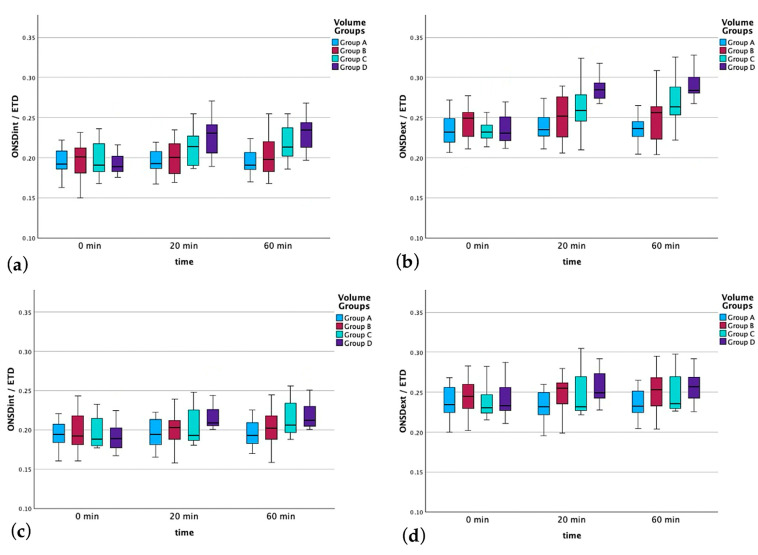
ONSDint/ETD and ONSDext/ETD ratios at baseline, 20 min, and 60 min after block across the four study groups. (**a**) Block-side ONSDint/ETD ratio. (**b**) Block-side ONSDext/ETD ratio. (**c**) Contralateral ONSDint/ETD ratio. (**d**) Contralateral ONSDext/ETD ratio. ONSD, optic nerve sheath diameter; ETD, eyeball transverse diameter.

On the block side, the ONSDint/ETD and ONSDext/ETD ratios increased markedly from baseline at 20 and 60 min in Groups C and D, while Group B showed only a modest increase in the ONSDext/ETD ratio and Group A showed no significant change (Figure 4a,b). On the contralateral side, both ratios increased significantly over time in Groups C and D, whereas Groups A and B showed no significant changes (Figure 4c,d). At 20 min, the median difference between Groups A and D was −0.049 (95% CI: −0.060 to −0.037) on the block side and −0.021 (95% CI: −0.039 to −0.007) on the contralateral side. At 60 min, the median difference was −0.049 (95% CI: −0.063 to −0.040) on the block side and −0.021 (95% CI: −0.034 to −0.006) on the contralateral side.

PI values increased markedly over time, particularly on the block side. Significant intergroup differences in PI were observed at 4, 6, and 8 min (*p* < 0.001) and persisted at 10 min (*p* = 0.002). In contrast, no significant intergroup differences were found at the remaining time points (0, 2, 15, 20, and 25 min; *p* > 0.05) (Table 3). On the nonblock side, significant intergroup differences in PI were found at baseline, 2, and 4 min (*p* = 0.046, *p* < 0.001, and *p* < 0.001). The time to reach threefold baseline PI (PI_3x_) differed significantly among groups, with shorter times observed as LA volume–dose increased (*p* < 0.001).

No significant intergroup differences were observed in EtCO_2_ values (*p* > 0.05). In intragroup time analysis, a significant change was observed only in the 30 mL group between 30 and 60 min (*p* = 0.011) (Table 4).

Sensory and motor block onset times decreased significantly with increasing LA volume–dose (*p* < 0.001). In pairwise comparisons, sensory block onset was significantly longer in Group A than in Groups B, C, and D (*p* < 0.001). Motor block onset was significantly longer in Group A than in Groups C and D, and in Group B than in Group D (*p* < 0.001). Analgesia duration increased significantly with increasing LA volume–dose. Analgesia duration was significantly shorter in Group A than in Groups B, C, and D, and in Group B than in Groups C and D (*p* < 0.001). No significant intergroup difference was found in supplemental LA requirement during surgery (*p* = 0.897). Median differences between Groups A and D were also observed for sensory block onset (+10 min; 95% CI: +5 to +10), motor block onset (+14 min; 95% CI: +9 to +15), and analgesia duration (−170 min; 95% CI: −215 to −125), with no significant difference between Groups C and D for analgesia duration (−2.5 min; 95% CI: −40 to +40). No clinically apparent complications were observed in any of the four groups throughout the study period (Table 5).

At 0, 1, 2, and 4 h, all groups reported a VPS of 0. No significant intergroup differences were observed at 6, 12, or 24 h (*p* > 0.05) (Table 6).

## 4. Discussion

In this study, we demonstrated that increasing LA volume–dose in US-guided SCBPB led to a significant increase in ONSD ratios. ONSD/ETD ratios increased significantly on both the block and contralateral sides in the 25 mL and 30 mL groups. No significant change was observed in the 15 mL group, and the 20 mL group showed only a limited increase in the ONSDext/ETD ratio on the block side. To our knowledge, this is the first prospective randomized study to evaluate the effects of LA volume–dose on ICP through ONSD measurement in SCBPB.

The groups were well balanced at baseline with respect to demographic and clinical characteristics, including age, BMI, sex, ASA classification, smoking status, and type and duration of surgery. Previous studies have reported that the ONSD/ETD ratio has been proposed as a more reliable parameter of elevated ICP than ONSD alone, as it accounts for interindividual variability in eyeball size [16,17,18]. Previous studies have suggested that ONSD/ETD ratios of approximately 0.22–0.25 may indicate elevated intracranial pressure, although these values vary depending on patient population and measurement techniques [16]. Therefore, no universally accepted cut-off has been established. In this study, we aimed to minimize individual differences by using the ratio of both ONSDint and ONSDext measurements to ETD. In our study, changes in ipsilateral and contralateral ONSD/ETD ratios were not statistically significant in Group A, which received the lowest volume. In contrast, Group B showed a unilateral increase only in the ONSDext/ETD ratio, whereas Groups C and D demonstrated significant bilateral increases in ONSD/ETD ratios. The bilateral increase in ONSD/ETD ratios, including on the contralateral side, suggests that the observed effects are not purely local. This finding may be attributed to systemic or centrally mediated mechanisms, such as autonomic responses, rather than direct spread of the local anesthetic. The relatively higher baseline ONSD/ETD values observed in our study population may be attributed to interindividual anatomical variability, measurement-related factors, and the absence of universally standardized threshold values. Previous studies have demonstrated that ONSD and ONSD/ETD measurements can vary considerably even among healthy individuals, and values approaching or exceeding proposed cut-off levels do not necessarily indicate pathological intracranial hypertension in the absence of clinical findings [19,20,21]. Therefore, the absence of symptoms in our study does not allow a definitive conclusion regarding the extent to which the observed increases in ONSD correspond to actual elevations in ICP. This finding further supports the notion that increases in ONSD/ETD ratios may not directly correspond to clinically significant intracranial hypertension. The relationship between ONSD changes and ICP elevation following SCBPB has not been sufficiently investigated in the literature. Although peripheral nerve blocks are generally considered to have minimal systemic effects, several studies have suggested that certain regional techniques, particularly those involving cervical or interscalene approaches, may influence intracranial dynamics. Gundogdu et al. reported an increase in ONSD in patients who received 25 mL interscalene block, and Kim et al. similarly demonstrated an increase in ONSD following 5 mL sympathetic ganglion block [9,12]. However, evidence remains limited and indirect, as most studies rely on surrogate markers rather than direct ICP measurements. In addition, the relationship between ONSD and intracranial pressure has been well established in previous studies using ocular ultrasonography in neurocritical care settings [19]. In the present study, no neurological symptoms suggestive of elevated intracranial pressure were observed in any patient.

Consistent with both studies, no clinical symptoms suggestive of elevated ICP were observed in our study. This suggests that intracranial compliance in healthy individuals may compensate for small increases in ICP. However, the lack of validation with an invasive gold-standard method for ICP measurement in our study limits the ability to definitively establish the relationship between increases in ONSD/ETD and ICP [22]. When absolute ONSD values and their changes from baseline are considered, the observed increases appear to be limited and do not reach the threshold values reported for intracranial hypertension. Although statistically significant differences were observed in the higher volume–dose groups, these changes may not reflect clinically meaningful elevations in intracranial pressure. In addition, variability in the measurements limits definitive clinical interpretation. The absence of significant changes in ONSD/ETD at the 15 mL volume–dose suggests that 15 mL may be a safer option than 25 or 30 mL in patients with reduced intracranial compliance who are at risk for elevated ICP. However, this interpretation should be made with caution, as patients with impaired intracranial compliance were not included in this study, ICP was not directly measured, and the observed ONSD changes did not reach levels that would suggest clinically significant intracranial hypertension. Furthermore, although increased ONSD/ETD values were observed in the 25 and 30 mL groups, no neurological symptoms developed in any patient. This finding indicates that these changes may not necessarily reflect clinically significant intracranial hypertension and that the clinical relevance of these findings remains uncertain. Accordingly, 25 and 30 mL volume–dose combinations should be carefully considered in patients with elevated ICP or those at risk.

The increase in PI is consistent with sympathetic blockade and vasodilation associated with peripheral nerve blocks, and this change has been shown to precede sensory and motor block findings [23,24]. The observed differences in perfusion index (PI) values measured from the contralateral extremity should be interpreted with caution. Although some intergroup differences were observed, the absence of significant intragroup changes over time suggests that a true sympathetic response did not occur in the contralateral extremity. In our study, it was also observed that PI values increased significantly in the early period, particularly on the blocked side, and that this increase became earlier and more pronounced as the LA volume–dose increased. The shortening of the time for PI to reach threefold its baseline value with increasing volume supports the premise that higher volumes produce a faster and more profound block. The disappearance of the difference among the groups at later time points indicates that the block effect reached a similar level over time. Although specific threshold values for PI have been defined in the literature, it has been reported that evaluating the increase relative to the baseline value and comparing it with the contralateral extremity is more reliable than using a single absolute value [25]. In the present study, although an early increase was observed on the contralateral side, the fact that PI did not reach threefold its baseline value suggests that a clinically significant sympathetic block developed exclusively on the blocked side.

In our study, no significant differences were detected among the groups in terms of EtCO_2_ values; a limited time-dependent increase was observed only in the 30 mL group, and these values remained within physiological limits. Kapoor et al. demonstrated a significant relationship between EtCO_2_ levels and ONSD [26]. However, it has been reported in the literature that the sensitivity of non-invasive EtCO_2_ measurement in reflecting PaCO_2_ changes is limited, particularly in spontaneously breathing patients [27]. The non-invasive measurement of EtCO_2_ in our study may have prevented the sensitive detection of CO_2_ changes; nevertheless, this can be interpreted as indicating that CO_2_ values measured within physiological limits do not create a difference in ONSD changes.

In our study, it was observed that as the LA volume–dose increased, the sensory and motor block onset times decreased. Similarly, the literature reports that high volumes accelerate block onset and prolong the duration of analgesia [28]. In the study by Bao et al., motor block onset was shorter in the high-volume group, whereas the sensory block onset rate did not differ between the groups [29]. However, some studies have found no significant relationship between volume and block onset time. Jeon et al. compared 20–35 mL mepivacaine volumes in US-guided SCBPB and found no significant difference among the groups in terms of onset time [30]. Similarly, when Schoenmakers et al. compared 15 mL and 40 mL of 1.5% mepivacaine in axillary brachial plexus block, block onset and efficacy were found to be similar, but it was demonstrated that the block duration was shortened by 17–19% with the lower volume [31]. In our study, high-volume administration was observed to increase the duration of analgesia, and there are data in the literature supporting this finding. Fredrickson et al. investigated the effect of ropivacaine volume and concentration on block duration in interscalene block and reported that increasing the volume of 0.375% ropivacaine from 10 mL to 40 mL prolonged the median block duration from approximately 10 h to 15 h [32]. Chadha et al., on the other hand, showed in a comparison between 20 mL and 35 mL of 0.5% ropivacaine in SCBPB that while onset times were similar, the analgesia duration was shortened by 21% in the low-volume group [33]. It is considered that these discrepancies may stem from variations in the concentration of the LA agents used, the total dose, and the level of block administration. Indeed, Fenten et al. reported in their study using mepivacaine in axillary brachial plexus block that the primary factor determining block duration is concentration and dose rather than volume [34]. In our findings, the shorter block onset and termination times in the low-volume–dose groups demonstrate that LA volume–dose is a significant determinant of block characteristics.

The similarity in postoperative VPS values between groups is consistent with the report of Bao et al., who observed no obvious analgesic differences between 20 mL and 30 mL of 0.375% ropivacaine in SCBPB [29]. This may be attributed to the standardized postoperative analgesic regimen of routine paracetamol with rescue tramadol.

Our study has several limitations. Its single-center design and limited sample size restrict the generalizability of the results. The inclusion of only low-risk patients within a specific age range limits the generalization of the findings to different patient populations. The effects of administering the same total dose of local anesthetic at different volumes were not evaluated in this study. Therefore, the observed effects cannot be attributed solely to volume. In addition, ICP was not measured using gold-standard invasive methods but was evaluated indirectly via ONSD; this limits the ability to definitively establish the relationship between an increase in ONSD and ICP. All ONSD measurements were performed by a single operator; therefore, interobserver variability could not be assessed. In addition, a formal intraobserver reliability analysis was not conducted, which may limit the reproducibility of the measurements. The fact that EtCO_2_ was measured using a non-invasive method and was not confirmed by arterial blood gas analysis or TcCO_2_ may have led to an insufficiently sensitive detection of CO_2_ changes. The fact that the measurements were obtained at specific time points may also have resulted in the failure to capture interim peak changes. Furthermore, although potential mechanisms such as phrenic nerve involvement, diaphragmatic dysfunction, altered ventilation, and impaired venous outflow were considered, these factors were not directly assessed in the present study. Specifically, phrenic nerve involvement and diaphragmatic function were not evaluated using ultrasound, ventilation changes were not confirmed by arterial blood gas or transcutaneous CO_2_ measurements, and venous flow dynamics were not assessed. Therefore, these proposed mechanisms should be interpreted with caution. It is considered that, in future studies, validation using invasive ICP monitoring or more sensitive CO_2_ measurement methods, as well as studies including different patient populations and using more frequent measurement intervals, would contribute to a clearer elucidation of this relationship.

## 5. Conclusions

In conclusion, increasing the LA volume–dose in US-guided SCBPB significantly accelerates sensory and motor block onset and prolongs block duration. The volume–dose-dependent increase in ONSD/ETD ratios observed on both the blocked and contralateral sides indicates that this effect may be associated with systemic or central mechanisms rather than being purely local. The early and pronounced increase in PI, particularly in high-volume–dose administrations, has been shown to rapidly and objectively reflect block success. However, the non-invasive measurement of EtCO_2_ may have precluded the detection of more subtle CO_2_ changes, limiting the full elucidation of the potential effects of physiological CO_2_ levels on ONSD. Collectively, these findings demonstrate that the LA volume–dose combinations used in SCBPB are associated with changes in block characteristics and in the US-derived ONSD/ETD ratios, which serve as indirect markers of ICP. While these findings indicate that LA volume–dose is an important determinant of both block characteristics and indirect ICP-related parameters such as ONSD/ETD ratios, they should be interpreted with caution. The study population consisted of relatively young ASA I–II patients without known intracranial pathology; therefore, the results may not be directly generalizable to patients with neurological disease, traumatic brain injury, impaired cerebral compliance, respiratory disease, or those at increased risk of intracranial hypertension.

## Figures and Tables

**Table 1:** Demographic and Clinical Characteristics.

Variable	Group A (n = 16)	Group B (n = 16)	Group C (n = 16)	Group D (n = 16)
Age, yr	30.9 ± 7.7	32.7 ± 9.0	35.3 ± 8.2	28.0 ± 9.9
BMI, kg/m^2^	25.0 ± 3.8	24.1 ± 4.3	27.3 ± 3.7	24.6 ± 3.1
Female sex, n (%)	7 (43.8)	5 (31.3)	8 (50.0)	4 (25.0)
ASA physical status, n (%)				
I	14 (87.5)	15 (93.8)	16 (100.0)	16 (100.0)
II	2 (12.5)	1 (6.3)	0 (0.0)	0 (0.0)
Comorbidities, n (%)				
Ankylosing spondylitis	1 (6.3)	0 (0.0)	0 (0.0)	0 (0.0)
Hepatitis B	1 (6.3)	0 (0.0)	0 (0.0)	0 (0.0)
Rheumatoid arthritis	0 (0.0)	1 (6.3)	0 (0.0)	0 (0.0)
Current smoker, n (%)	7 (43.8)	5 (31.3)	7 (43.8)	9 (56.3)
Type of surgery, n (%)				
Elbow	2 (12.5)	1 (6.3)	1 (6.3)	0 (0.0)
Forearm	1 (6.3)	2 (12.5)	1 (6.3)	0 (0.0)
Wrist	1 (6.3)	2 (12.5)	2 (12.5)	3 (18.8)
Hand	12 (75.0)	11 (68.8)	12 (75.0)	13 (81.3)
Operated side, n (%)				
Right	11 (68.8)	10 (62.5)	11 (68.8)	9 (56.3)
Left	5 (31.3)	6 (37.5)	5 (31.3)	7 (43.8)
Duration of surgery, min	64.8 ± 20.4	75.5 ± 25.8	66.6 ± 27.7	60.6 ± 27.1

Data are presented as mean ± SD or n (%). ASA, American Society of Anesthesiologists; BMI, body mass index.

**Table 2:** ONSDint/ETD and ONSDext/ETD Ratios (mean ± SD).

Groups		Time (min)	Group A	Group B	Group C	Group D	*p*
Block Side	ONSDint/ETD	0.	0.196 ± 0.016	0.198 ± 0.022	0.211 ± 0.056	0.196 ± 0.021	0.898
		20.	0.198 ± 0.018	0.200 ± 0.021	0.213 ± 0.022 *	0.226 ± 0.022 *	0.002
		60.	0.194 ± 0.016	0.202 ± 0.025	0.219 ± 0.023 *^,†^	0.227 ± 0.029 *^,†^	0.001
	*p*		0.410	0.334	0.008	0.001	
	ONSDext/ETD	0.	0.235 ± 0.019	0.240 ± 0.025	0.237 ± 0.022	0.237 ± 0.022	0.870
		20.	0.239 ± 0.020	0.249 ± 0.028 *	0.262 ± 0.029 *	0.288 ± 0.018 *	<0.001
		60.	0.237 ± 0.015	0.250 ± 0.030 *^,†^	0.270 ± 0.029 *^,†^	0.286 ± 0.029 *^,†^	<0.001
	*p*		0.125	0.015	<0.001	<0.001	
Contralateral Side	ONSDint/ETD	0.	0.195 ± 0.016	0.198 ± 0.023	0.196 ± 0.018	0.194 ± 0.023	0.868
		20.	0.196 ± 0.017	0.201 ± 0.020	0.204 ± 0.022 *	0.218 ± 0.020 *	0.001
		60.	0.197 ± 0.016	0.201 ± 0.022	0.215 ± 0.022 *^,†^	0.220 ± 0.020 *^,†^	0.001
	*p*		0.173	0.460	0.002	0.001	
	ONSDext/ETD	0.	0.238 ± 0.020	0.244 ± 0.021	0.239 ± 0.024	0.240 ± 0.022	0.754
		20.	0.233 ± 0.020	0.247 ± 0.021	0.247 ± 0.027 *	0.256 ± 0.019 *	<0.001
		60.	0.237 ± 0.019	0.251 ± 0.026	0.249 ± 0.024 *^,†^	0.257 ± 0.017 *^,†^	<0.001
	*p*		0.245	0.088	0.013	<0.001	

ETD, eyeball transverse diameter; ONSDint, internal; ONSDext, external optic nerve sheath diameter. * Significantly different from baseline (0 min); ^†^ significantly different from 20 min (Wilcoxon signed-rank test). Intergroup comparisons by Kruskal–Wallis test; intragroup comparisons by Friedman test.

**Table 3:** PI Values (mean ± SD).

Groups	PI (min)	Group A	Group B	Group C	Group D	*p*
Block side	0.	2.53 ± 1.59	2.81 ± 1.14	2.99 ± 1.34	2.46 ± 1.51	0.659
	2.	2.48 ± 1.37	2.95 ± 1.3	3.45 ± 1.82	4.34 ± 2.64	0.063
	4.	2.81 ± 1.51	3.22 ± 1.22	5.3 ± 3.08	7.57 ± 3.2	<0.001
	6.	3.38 ± 1.92	4.12 ± 1.39	7.79 ± 4.07	9.33 ± 2.61	<0.001
	8.	4.22 ± 2.53	6.11 ± 2.74	9.31 ± 4.26	10.99 ± 3.62	<0.001
	10.	6.07 ± 3.38	9.57 ± 4.08	10.48 ± 3.96	10.68 ± 3.78	0.002
	15.	8.81 ± 4.69	10.99 ± 3.46	10.5 ± 3.59	10.65 ± 3.26	0.394
	20.	10.51 ± 6	12.34 ± 2.92	11.43 ± 3.94	10.9 ± 3.95	0.671
	25.	10.44 ± 5.98	12.19 ± 2.77	11.17 ± 3.64	10.85 ± 3.96	0.481
PI_3x_		14.56 ± 5.45	11.94 ± 3.86	7.06 ± 2.82	4.5 ± 1.86	<0.001
Nonblock side	0.	2.02 ± 0.97	2.75 ± 1.05	3.76 ± 2.93	3.05 ± 1.81	0.046
	2.	2 ± 0.86	2.75 ± 1.17	4.36 ± 3.51	3.66 ± 1.3	<0.001
	4.	2 ± 0.93	2.85 ± 1.07	4.35 ± 3.1	3.64 ± 1.38	<0.001
	6.	2.34 ± 1.03	2.98 ± 0.9	4.45 ± 3.04	3.69 ± 1.53	0.008
	8.	2.45 ± 1.09	3 ± 1.04	4.73 ± 2.96	3.88 ± 1.56	0.003
	10.	2.55 ± 1.21	3 ± 1.03	4.59 ± 2.98	3.8 ± 1.57	0.006
	15.	2.62 ± 1.08	3.06 ± 1.13	5.24 ± 2.61	3.42 ± 1.15	0.001
	20.	2.74 ± 1.22	3.21 ± 1.5	5.14 ± 2.6	3.65 ± 1.23	0.003
	25.	2.79 ± 1.23	3.22 ± 1.42	5.56 ± 2.66	3.67 ± 1.17	0.002
PI_3x_		-	-	-	-	

PI_3x_: Time to threefold baseline PI on the block side (min).

**Table 4:** EtCO_2_ (mmHg) (mean ± SD).

Groups	Group A	Group B	Group C	Group D	*p*
0 min	35.31 ± 1.58	34.5 ± 1.79	35.5 ± 1.67	35.75 ± 2.77	0.089
10 min	36.06 ± 2.54	34.44 ± 1.82	35.81 ± 2.43	36.06 ± 2.84	0.071
20 min	35.81 ± 2.54	34.56 ± 1.67	35.75 ± 1.44	35.88 ± 1.82	0.082
30 min	36.44 ± 2.39	34.94 ± 2.05	36 ± 1.21	35.63 ± 1.67	0.267
60 min	36.63 ± 1.89	35.69 ± 1.89	36.06 ± 1.18	37 ± 2.31 *	0.116
Post op	36.75 ± 1.48	35.5 ± 1.03	36 ± 1.75	36.88 ± 1.75	0.053
*p*	0.111	0.323	0.445	0.011	

EtCO_2_, end-tidal carbon dioxide; Post op, Measurement in the postoperative unit. * Significantly different from 30 min (Wilcoxon signed-rank test). Intragroup comparisons by Friedman test.

**Table 5:** Sensory and Motor Block Onset Times, Analgesia Duration, Supplemental LA Requirement, and Perioperative Complications.

Groups	Group A	Group B	Group C	Group D	*p*
Sensory block onset (min)	13.75 ± 3.41	10.34 ± 2.72	9.38 ± 3.10	7.19 ± 2.56	<0.001
Motor block onset (min)	20.63 ± 6.55	15.63 ± 3.09	12.50 ± 4.08	9.69 ± 4.99	<0.001
Analgesia duration (min)	535 ± 73.89	594.06 ± 51.06	716.25 ± 64.77	712.50 ± 51.28	<0.001
Supplemental LA requirement, n (%)	2 (12.5)	1 (6.3)	1 (6.3)	0 (0)	0.897
Supplemental LA amount (mL)	0.88 ± 2.42	0.38 ± 1.50	0.38 ± 1.50	0	0.107
Perioperative complications, n (%)	0 (0)	0 (0)	0 (0)	0 (0)	–

**Table 6:** Postoperative Verbal Pain Score (VPS) (mean ± SD).

Time (h)	Group A	Group B	Group C	Group D	*p*
0	0	0	0	0	–
1	0	0	0	0	–
2	0	0	0	0	–
4	0	0	0	0	–
6	0.25 ± 1.00	0	0	0	0.411
12	5.44 ± 1.75	5.63 ± 1.40	3.75 ± 3.13	3.50 ± 2.80	0.079
24	2.94 ± 1.23	2.50 ± 1.03	3.25 ± 1.52	3.88 ± 1.40	0.054

VPS, Verbal Pain Score. Intergroup comparisons by Kruskal–Wallis test.

## Data Availability

The trial protocol, statistical analysis plan, and de-identified participant data presented in this study are available from the corresponding author upon reasonable request.

## Supplementary Materials

The following supporting information can be downloaded at: https://www.mdpi.com/article/10.3390/medicina62061103/s1, Table S1. Absolute ONSD Values (cm) (mean ± SD).

## Abbreviations

The following abbreviations are used in this manuscript:

ASA	American Society of Anesthesiologists
BMI	Body mass index
ETD	Eyeball transverse diameter
EtCO_2_	End-tidal carbon dioxide
ICP	Intracranial pressure
LA	Local anesthetic
ONSD	Optic nerve sheath diameter
ONSDext	External optic nerve sheath diameter
ONSDint	Internal optic nerve sheath diameter
PaCO_2_	Partial pressure of arterial carbon dioxide
PI	Perfusion index
SCBPB	Supraclavicular brachial plexus block
SpO_2_	Peripheral oxygen saturation
TcCO_2_	Transcutaneous carbon dioxide
US	Ultrasound
