# Supplementary material for: Association Between Local Anesthetic Volume–Dose Combinations and Optic Nerve Sheath Diameter as an Indirect Marker of Intracranial Pressure During Ultrasound-Guided Supraclavicular Brachial Plexus Block: A Randomized Trial
Source: Medicina (Kaunas). 2026 Jun 5;62(6):1103. doi: 10.3390/medicina62061103 (PMC13303052; doi:10.3390/medicina62061103)
Supplement: Supplementary file 1 [file medicina-62-01103-s001.zip › Supplementary Table S1.pdf]

Supplementary Table S1. Absolute ONSD Values (cm) (mean ± SD)

Groups		Time (min)	Group A	Group B	Group C	Group D	*p*
Block side	Transverse ONSDint	0.	0.44±0.04	0.44±0.05	0.44±0.05	0.45±0.05	0.930
		20.	0.44±0.04	0.45±0.05*	0.48±0.05*	0.52±0.06*	**0.002**
		60.	0.44±0.04	0.46±0.05*	0.5±0.05*,†	0.53±0.05*,†	**<0.001**
	p		0.677	**0.008**	**<0.001**	**<0.001**	
	Sagittal ONSDint	0.	0.44±0.04	0.44±0.05	0.45±0.05	0.44±0.05	0.968
		20.	0.45±0.04	0.45±0.04	0.48±0.05	0.52±0.05*	**0.001**
		60.	0.44±0.04	0.45±0.05*	0.49±0.05*,†	0.53±0.04*	**<0.001**
	p		0.749	**0.023**	**<0.001**	**<0.001**	
	Mean ONSDint	0.	0.45±0.04	0.44±0.04	0.48±0.13	0.45±0.05	0.964
		20.	0.45±0.04	0.45±0.05	0.48±0.05*	0.52±0.05*	**0.001**
		60.	0.44±0.04	0.46±0.06*,†	0.5±0.05*,†	0.53±0.04*,†	**<0.001**
	p		0.322	**0.028**	**0.001**	**<0.001**	
	Transverse ONSDext	0.	0.54±0.05	0.54±0.05	0.54±0.05	0.55±0.05	0.924
		20.	0.54±0.04	0.57±0.06*	0.6±0.07	0.66±0.03*	**<0.001**
		60.	0.54±0.04	0.57±0.06*	0.61±0.07*,†	0.68±0.03*,†	**<0.001**
	p		0.472	**0.002**	**0.001**	**<0.001**	
	Sagittal ONSDext	0.	0.54±0.05	0.53±0.04	0.53±0.05	0.54±0.06	0.995
		20.	0.54±0.04	0.56±0.05	0.61±0.07*	0.66±0.05*	**<0.001**
		60.	0.54±0.03	0.56±0.06*,†	0.62±0.08*,†	0.66±0.05*,†	**<0.001**
	p		0.352	**0.001**	**0.001**	**<0.001**	
	Mean ONSDext	0.	0.54±0.04	0.54±0.04	0.54±0.05	0.54±0.05	0.999
		20.	0.54±0.04	0.56±0.06	0.6±0.07*	0.66±0.04*	**<0.001**
		60.	0.54±0.04	0.57±0.07	0.61±0.07*	0.67±0.04*,†	**<0.001**
	p		0.208	0.08	**0.001**	**<0.001**	
Contralateral side	Transverse ONSDint	0.	0.44±0.04	0.44±0.05	0.44±0.05	0.44±0.05	0.929
		20.	0.45±0.04	0.45±0.04	0.46±0.05	0.50±0.04*	**<0.001**
		60.	0.45±0.04	0.45±0.05	0.49±0.05*,†	0.51±0.04*	**<0.001**
	p		0.415	0.322	**<0.001**	**<0.001**	
	Sagittal ONSDint	0.	0.44±0.04	0.44±0.05	0.44±0.04	0.44±0.04	0.979
		20.	0.45±0.04	0.45±0.04	0.47±0.05	0.49±0.05*	**<0.001**
		60.	0.45±0.04	0.46±0.05	0.49±0.05*,†	0.50±0.04*	**<0.001**
	p		0.203	0.178	**<0.001**	**<0.001**	
	Mean ONSDint	0.	0.45±0.04	0.44±0.05	0.44±0.05	0.44±0.05	0.901
		20.	0.45±0.04	0.45±0.04	0.46±0.05	0.50±0.04*	**<0.001**
		60.	0.45±0.04	0.46±0.05	0.49±0.05*,†	0.50±0.04*	**<0.001**
	p		0.061	0.203	**<0.001**	**<0.001**	
	Transverse ONSDext	0.	0.54±0.05	0.55±0.04	0.55±0.06	0.55±0.05	0.959
		20.	0.53±0.04	0.56±0.04	0.56±0.06	0.59±0.04*	**<0.001**
		60.	0.54±0.04	0.57±0.05	0.56±0.06*,†	0.59±0.04*	**<0.001**
	p		0.193	0.093	**0.001**	**<0.001**	
	Sagittal ONSDext	0.	0.54±0.05	0.54±0.04	0.53±0.05	0.55±0.05	0.758
		20.	0.54±0.04	0.55±0.05	0.55±0.06	0.58±0.04*	**<0.001**
		60.	0.54±0.05	0.56±0.05*	0.56±0.05*,†	0.58±0.04*	**<0.001**
	p		0.342	**0.003**	**0.001**	**<0.001**	
	Mean ONSDext	0.	0.54±0.05	0.54±0.04	0.54±0.06	0.55±0.04	0.898
		20.	0.53±0.04	0.56±0.04	0.56±0.06	0.58±0.04*	**<0.001**
		60.	0.54±0.04	0.57±0.05	0.56±0.05*,†	0.59±0.04*	**<0.001**
	p		0.322	0.172	**0.001**	**<0.001**	

ONSD, optic nerve sheath diameter; ONSDint, internal optic nerve sheath diameter; ONSDext, external optic nerve sheath diameter. * Significantly different from baseline (0 min); † significantly different from 20 min (Wilcoxon signed-rank test). Intergroup comparisons by Kruskal–Wallis test; intragroup time comparisons by Friedman test.
